# The pandemic of childhood obesity: Challenges and possibilities from physical activity

**DOI:** 10.34172/hpp.2022.29

**Published:** 2022-12-10

**Authors:** Alejandro Almonacid-Fierro, Javier González-Almonacid

**Affiliations:** ^1^Department of Physical Activity Sciences, Faculty of Educational Sciences, Universidad Católica del Maule, Talca, Chile; ^2^Traumatology Service, Hospital San Borja Arriarán, Santiago, Chile; ^3^School Emergency Unit, Clínica Alemana de Santiago, Chile

## Dear Editor,

 The dramatic increase observed in the prevalence and severity of childhood obesity has important implications for morbidity and mortality during adulthood, consequently, immediate measures should be taken to prevent excess weight during childhood, as primary prevention, and to treat children and adolescents who are already overweight.^1^ Some of the consequences of obesity are related to an increased risk of chronic non-communicable diseases (NCDs). In addition, being overweight is directly related to elevated plasma insulin concentrations, altered lipid profile, and hypertension.^2^ This incidence can have an impact on life expectancy, affecting growth and musculoskeletal development.

 The World Health Organization (WHO), states that obesity is one of the most serious health problems we have to face as a planetary society, such that, by 2025, it is estimated that 2.3 billion adults worldwide will be overweight, with 700 million individuals with obesity.^3^ Etiologically, obesity is considered multifactorial, as it interacts with genetic, metabolic, nutritional, psychosocial, and environmental factors and lifestyle changes. It can also be associated with genetic syndromes or metabolic endocrine disorders. Obesity is broadly defined as an excess body fat mass, characterized by a chronic inflammatory state and excessive accumulation of body fat.^4^

 Studies point out that modernity, together with an increasingly sedentary lifestyle and a diet based on ultra-processed foods, brings with it a social impact from early childhood, as can observed in the increase in the number of cases in obese children. Thus, the social distancing resulting from the COVID-19 pandemic has exacerbated these factors.^5^ In the current scenario, there is a growing increase in the risk factors for childhood obesity, such as poor nutrition from early childhood, sedentary lifestyles, and increased use of screens in general.^1^ This reality has already been observed with the increasing modernity and advancement of technology; however, the current pandemic caused by COVID-19 has further aggravated circumstances, enhancing the described risk factors for obesity and making the child population more susceptible to this development.^6^

 Many factors contribute to the childhood obesity epidemic, such as genetics, unhealthy habits, lack of physical activity (PA), and environmental difficulties. However, the practice of PA in the fight against obesity during childhood and adolescence can contribute in three ways: I) PA in this phase helps to steady the energy balance; II) active youth tend to become active adults; III) active youth are less likely to develop obesity and its comorbidities in adulthood, while inactive youth have more than 90% chance of becoming sedentary adults^7^. Moreover, the benefits of PA go beyond the control of obesity, constituting an important component of the prevention and treatment of coronary heart disease, hypertension, musculoskeletal diseases, and respiratory diseases.^7^

 The biological effects related to elevated PA levels in children are lower blood pressure, more favorable serum lipid and lipoprotein levels, more insulin sensitivity, and less adipose tissue accumulation, as it is also considered an important factor in achieving and maintaining adequate bone strength, contributing to normal skeletal development.^8^ Since obesity is considered an inflammatory disease and physical exercise directly modulates such processes, it is essential to implement physical exercise programs to improve the inflammatory response in obese children and adolescents. The PA recommendations used by the American College of Sports Medicine for children aged 6-12 years are at least 60 minutes of physical exercise daily, and activities of a vigorous nature three days a week.^9^ Consequently, it is urgent to implement various measures to achieve these standards, with systemic actions by the State and the families themselves to address the pandemic of childhood obesity.

## Acknowledgements

 The authors appreciate the reviewers for their insightful comments.

## Author Contributions

**Conceptualization: **Alejandro Almonacid-Fierro.

**Formal Analysis:** Alejandro Almonacid-Fierro, Javier González-Almonacid.

**Writing – original draft:** Alejandro Almonacid-Fierro, Javier González-Almonacid.

**Writing – review & editing: **Alejandro Almonacid-Fierro, Javier González-Almonacid.

## Funding

 No funding received.

## Ethical Approval

 This study does not require any approval from the responsible ethics committee as it used secondary data.

## Competing Interests

 None.
